# Laparoscopic liver resection in the semiprone position for tumors in the anterosuperior and posterior segments, using a novel dual-handling technique and bipolar irrigation system

**DOI:** 10.1007/s00464-014-3469-y

**Published:** 2014-03-13

**Authors:** Tetsuo Ikeda, Takao Toshima, Norifumi Harimoto, Youichi Yamashita, Toru Ikegami, Tomoharu Yoshizumi, Yuji Soejima, Ken Shirabe, Yoshihiko Maehara

**Affiliations:** Department of Surgery and Science, Graduate School of Medical Sciences, Kyushu University, 3-1-1 Maidashi, Higashi-ku, Fukuoka, 812-8582 Japan

**Keywords:** Pure laparoscopic hepatectomy, Semiprone position, Anterosuperior and posterior segments, Dual-handling technique, Intercostal transthoracic port

## Abstract

**Background:**

Hepatic tumors in the lower edge and lateral segments are commonly treated by laparoscopic liver resection. Tumors in the anterosuperior and posterior segments are often large and locally invasive, and resection is associated with a higher risk of insufficient surgical margins, massive intraoperative bleeding, and breaching of the tumor. Laparoscopic surgery for such tumors often involves major hepatectomy, including resection of a large volume of normal liver tissue. We developed a novel method of laparoscopic resection of tumors in these segments with the patient in the semiprone position, using a dual-handling technique with an intercostal transthoracic port. The aim of this study was to evaluate the safety and usefulness of our technique.

**Methods:**

Of 160 patients who underwent laparoscopic liver resection at our center from June 2008 to May 2013, we retrospectively reviewed those with tumors in the anterosuperior and posterior segments. Patients were placed supine or semilateral during surgery until January 2010 and semiprone from February 2010.

**Results:**

Before the introduction of the semiprone position in February 2010, a total of 7 of 40 patients (17.5 %) with tumors in the anterosuperior and posterior segments underwent laparoscopic liver resection, and after introduction of the semiprone position, 69 of 120 patients (57.5 %) with tumors in the anterosuperior and posterior segments underwent laparoscopic liver resection (*P* < 0.001). There were no conversions to open surgery, reoperations, or deaths. The semiprone group had a significantly higher proportion of patients who underwent partial resection or segmentectomy of S7 or S8, lower intraoperative blood loss, and shorter hospital stay than the supine group (all *P* < 0.05). Postoperative complication rates were similar between groups.

**Conclusions:**

Laparoscopic liver resection in the semiprone position is safe and increases the number of patients who can be treated by laparoscopic surgery without increasing the frequency of major hepatectomy.

**Electronic supplementary material:**

The online version of this article (doi:10.1007/s00464-014-3469-y) contains supplementary material, which is available to authorized users.

The first laparoscopic nonanatomical liver resection for focal nodular hyperplasia was reported by Gagner et al. [1]. Since then, improvements in laparoscopic instruments have significantly improved the safety of laparoscopic liver resection [2–9]. However, laparoscopic nonanatomical resection generally is performed only for tumors located in the lower edge and lateral segments (Couinaudrs segments S2, S3, S4b, S5, and S6), because the posterosuperior segments (S1, S4a, S7, and S8) are difficult to visualize and beyond the reach of the surgical instruments. Nonanatomical partial resection and anatomical minor resection (S6, S7, or S8 segmentectomy) preserve liver parenchyma and are less invasive than right hemihepatectomy. Tumors in the posterosuperior segments are usually are resected by open surgery, which is much more invasive than laparoscopic surgery and leaves a large wound.

We developed a novel method for laparoscopic nonanatomical resection of tumors located in the right portions of S1, S6, S7, and S8. This includes the anterosuperior and posterior areas of the liver, except S4a but plus S6, and represents almost half of the liver volume.

Some high-volume centers have reported that laparoscopic resection of the posterosuperior segments can be performed as safely as resection of the anterolateral segments by an experienced surgeon [10, 11]. However, few specific techniques have been described. Reports indicate that patients with tumors of the posterosuperior segments (S1, S7, S8, and S4a) are more likely to undergo hemihepatectomy and less likely to undergo nonanatomical resection or segmentectomy than patients with tumors of the anterolateral segments (S2, S3, S4b, S5, and S6) [12, 13].

The aim of this study was to retrospectively evaluate the outcomes of laparoscopic liver resection in the semiprone position in patients with tumors in the anterosuperior segment (S8), posterior segments (S6 and S7), and parts of the caudate lobe (caudate process and paracaval portion of S1) compared with outcomes of resection in the conventional supine position [14, 15].

## Methods

### Patients

**Fig. 1:**
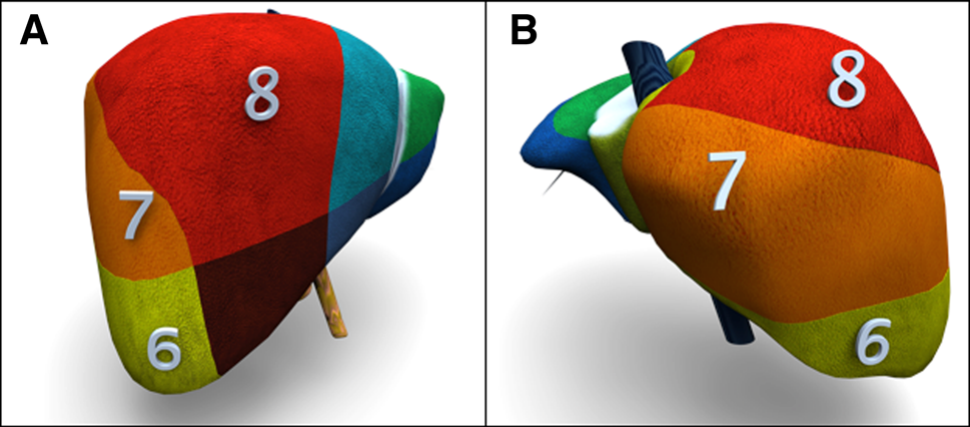
Illustration of liver segments. Patients who underwent laparoscopic resection of malignant tumors of the anterosuperior segment (S8), posterosuperior segment (S7), posteroinferior segment (S6), and right superior portion of the caudate lobe (S1) were included in this study. **a** Right anterior view. **b** Right posterior view

A total of 160 patients underwent laparoscopic resection of liver tumors at our center between June 2008 and May 2013. Of these, 76 patients had tumors located in the anterosuperior or posterior segments. The first 20 of these 76 patients underwent surgery in the supine position. Patients were carefully positioned according to tumor location and patient habitus; in some cases the right side of the patient was tilted upward by up to 45°. The first laparoscopic partial hepatectomy in the semiprone position was performed in February 2010 in a patient with a tumor in S7. Until October 2011, we performed laparoscopic liver resection on patients in the semiprone position only for tumors in S6, S7, and the posterior portion of S8. We now perform laparoscopic liver resection in the semiprone position for tumors in all parts of S6, S7, and S8 and the right portion of S1 (Fig. 1).

The indications for laparoscopic liver resection are similar to those for open liver resection with respect to preoperative assessment of liver function, type of liver resection, and postoperative care. However, patients with tumors >4 cm in diameter, tumors invading or adjacent to the main portal pedicle or inferior vena cava (IVC), or tumors adjacent to the main hepatic veins were excluded. The type of resection was determined based on the depth of lesions, number of lesions, locations of lesions relative to major vascular structures, and hepatic functional reserve. Major liver resection, including right hepatectomy, right posterior sectionectomy, or left hepatectomy, was considered in patients with a deep tumor when the remaining liver function was expected to be adequate. For metastatic liver tumors from colorectal cancer, liver resection was performed when there was no evidence of extrahepatic disease.

Standard preoperative investigations included routine abdominal spiral computed tomography (CT) and contrast ultrasonography, abdominal magnetic resonance imaging, and positron emission tomography if required; chest X-ray or CT; and serum biochemistry testing. To determine the operative method (laparoscopic or open) and extent of resection, all patients underwent preoperative assessment of liver functional reserve with liver function testing, Child-Pugh classification, and indocyanine green retention rate at 15 min.

### Laparoscopic liver resection in the semiprone position

The patient was placed in the semiprone position, which is similar to the position while breathing during front crawl swimming. The surgeon was positioned on the left cranial side of the patient, and the camera operator was positioned next to the surgeon on the left side of the patient.

**Fig. 2:**
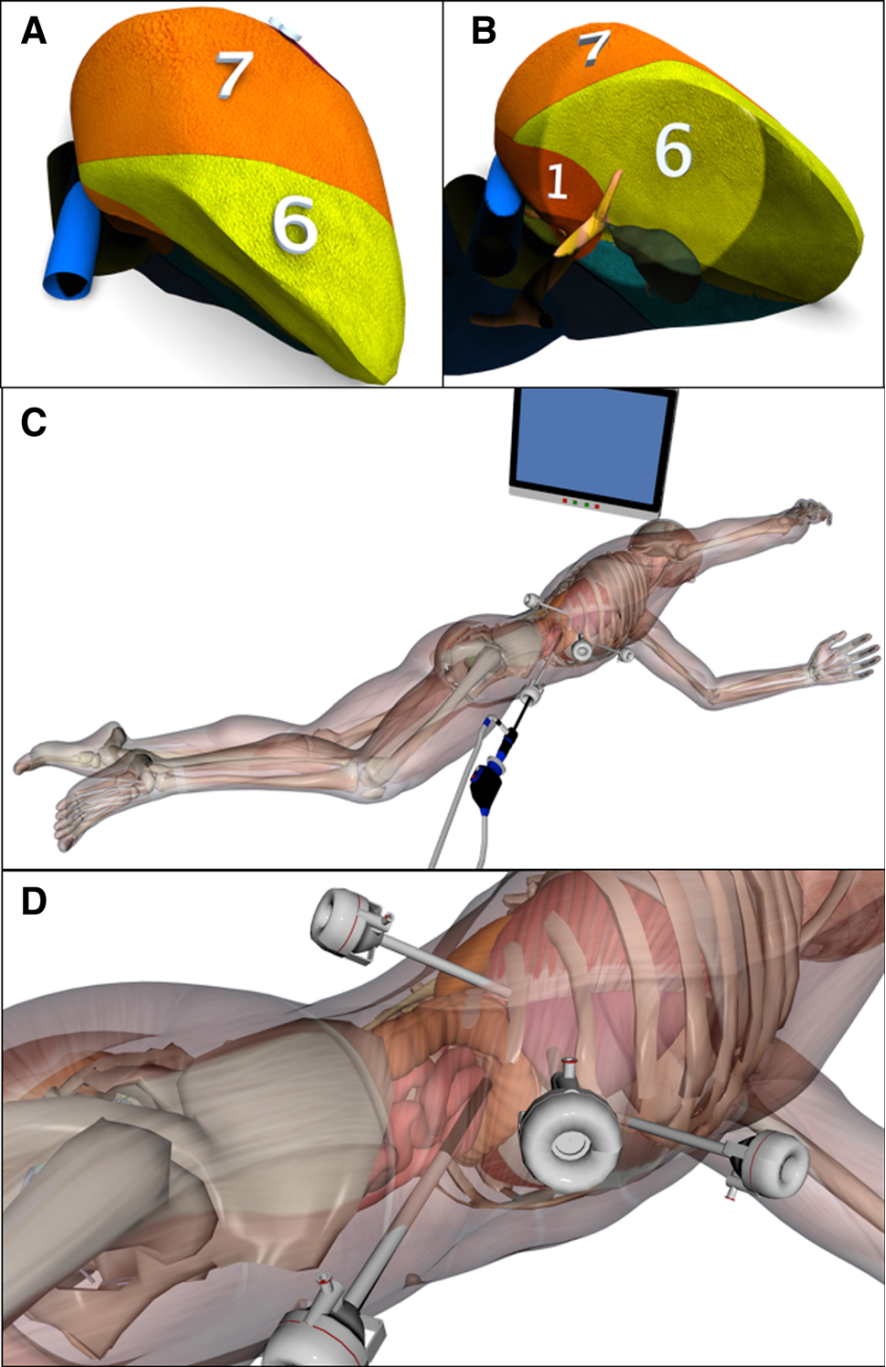
Laparoscopic liver resection in the semiprone position for tumors in the posteroinferior segment (S6) and right inferior portion of the caudate lobe (S1). **a** Right posterior view immediately after inserting the laparoscope. **b** Right inferior view when the lower surface of S6 is rising to the ventral side. **c** Semiprone position during surgery. **d** Port sites: one port was placed in the right pararectal line 10 cm below the subcostal margin for the camera, and three trocars were inserted through ports below the subcostal margin in the right pararectal line, anterior axillary line, and posterior axillary line

The port sites for resection of S6 and the right inferior portion of S1, including the caudate process, are shown in Fig. 2. The first port was placed in the right pararectal line, 10 cm below the costal margin, and was used to introduce a 30° laparoscope. Three trocars were placed below the costal margin in the right pararectal line, anterior axillary line, and posterior axillary line.

**Fig. 3:**
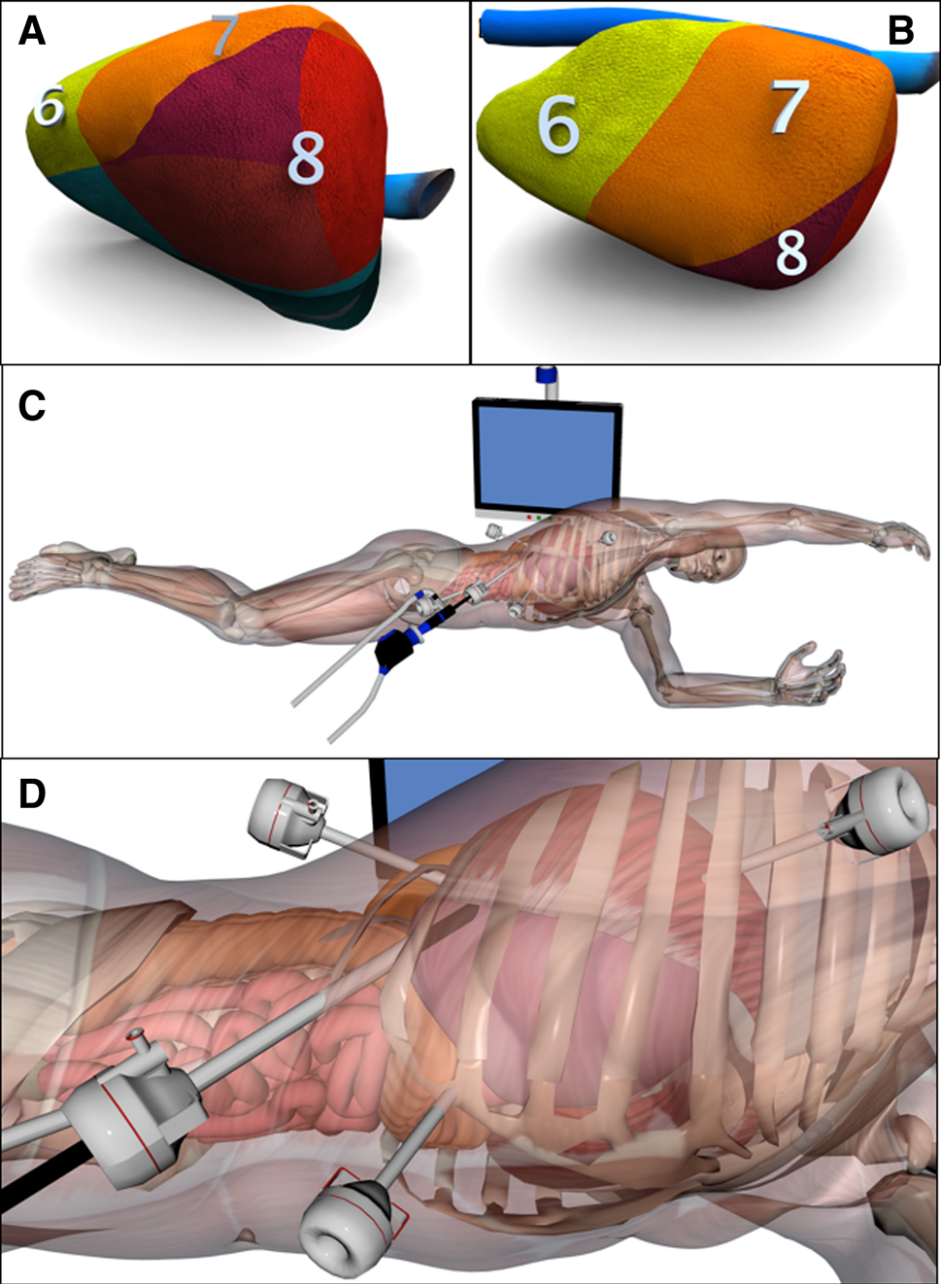
Laparoscopic liver resection in the semiprone position for tumors in the posterosuperior segment (S7), anterosuperior segment (S8), and right superior portion of the caudate lobe (S1). **a** Right anterior view before the right triangular and coronary ligaments are divided. **b** Right anterior view after the right triangular and coronary ligaments are divided. **c** Semiprone position during surgery. The patient position is almost the same as in Fig. 2. As the surgeon stands on the cranial side to use the intercostal port, the left hand of the patient is moved towards the head. **d** Port sites: an additional intercostal port was inserted at the seventh intercostal space in the anterior axillary line

For resection of S7, S8, and the right superior portion of S1, the laparoscope and ports initially were placed as described above. After hilar dissection and mobilization of the liver, an intercostal port was inserted at about the seventh intercostal space in the anterior axillary line (Fig. 3). The camera was inserted in the anterior axillary line, below the costal margin if necessary. Differential lung ventilation was used when intercostal ports were planned.

Although this patient position is stable, a vacuum mattress and two backboards were used to control rotation and for safety. A carbon dioxide pneumoperitoneum was established and was maintained at 8–15 mmHg. When necessary, such as for parenchymal dissection between the anterior segment and the internal portion of the liver during anterior sectionectomy, S8 segmentectomy, and right hemihepatectomy, the patient was rotated by tilting the left side of the operating table downward by up to 20°–30°. The patient stayed in the semiprone position throughout the procedure (see Video 1, which demonstrates patient position and port sites).

To perform hilar dissection, Rouviere’s sulcus (a fissure on the liver to the right of the hilum between S6 and S5) was oriented. The portal pedicles of S6 and the posterior and anterior segments were separated. The portal pedicle of S7 could be visualized after the portal pedicle of S6 was divided. The portal pedicles of segments with tumor visualized on preoperative imaging were divided so that the ischemic area corresponded to the tumor location (see Video 2, which demonstrates hilar dissection).

The right triangular and coronary ligaments were partially divided. The right inferior hepatic vein and short hepatic vein were also carefully divided. During liver mobilization, the position of the right lobe could be controlled by an assistant using only a 5-mm pledget (see Video 3, which demonstrates mobilization of the right lobe).

The surface of the liver was divided using mainly bipolar scissors fitted with a silicone tube dripping saline to the tip, and the liver parenchyma was transected using bipolar scissors or bipolar forceps such as the BiClamp™ (ERBE, Germany) or LigaSure™ (Covidien, Mansfield, MA) fitted with a saline drip. If parenchymal division of S7, S8, or the right superior portion of S1 was needed, left one-lung ventilation was initiated and an intercostal port was placed, using a balloon to isolate the chest from the abdominal cavity. This port was used by the right hand of the operator (see Video 4, which demonstrates the dual-handling technique of an intercostal port for division of the parenchyma during right hemihepatectomy). The surgical techniques of right hemihepatectomy and posterior sectionectomy in the semiprone position have previously been described in detail [14, 15]. The resected specimen was placed in a plastic bag and extracted through the right lateral trocar site, which was enlarged as needed.

Major resection was defined as hemihepatectomy or right posterior sectionectomy, and minor resection was defined as segmentectomy or tumorectomy. Tumors were defined as deep if they were located >2 cm from the liver surface on preoperative CT.

### Laparoscopic liver resection in the supine or semilateral position

Twenty patients underwent laparoscopic liver resection in the supine position. Patient position was carefully adjusted according to tumor location and patient habitus; if necessary, the right side of the patient was tilted upward by up to 45°. The surgical technique has previously been described in detail [5].

### Statistical analysis

Outcomes were compared between the supine and semiprone groups. Data are presented as mean (range) or number (percentage). Differences between groups were analyzed using JMP 5.1 software with Fisher’s exact test or *χ* ^2^ test as appropriate. Continuous variables were compared using Student’s *t* test.

## Results

Before the introduction of the semiprone position in February 2010, a total of 7 of 40 patients (17.5 %) with tumors in the anterosuperior and posterior segments underwent laparoscopic liver resection, and after the introduction of the semiprone position, 69 of 120 patients (57.5 %) with tumors in the anterosuperior and posterior segments underwent laparoscopic liver resection. (*P* < 0.001).

The indications for laparoscopic liver resection are given in Table 1. The majority of liver tumors in both groups were hepatocellular carcinoma (HCC).

**Table 1:** Indications for laparoscopic liver resection

Type of tumor	Group-S (*n* = 20)	Group-SP (*n* = 56)	*P*	Total (*n* = 76)
Primary liver tumor	15	30	NS	45
Hepatocellular carcinoma	15	29		44
Cholangiocarcinoma	0	1		1
Metastatic tumor	5	26	NS	31
Colorectal adenocarcinoma	4	26		30
Gastric carcinoid tumor	1	0		1

*Group-S* supine patients, *Group-SP* semiprone patients, *NS* not significant

The preoperative characteristics of the patients are listed in Table 2. Mean patient age, sex, body mass index, history of laparotomy, and preoperative chemotherapy were similar between the two groups. Most patients had underlying liver disease due to hepatitis B or C virus infection or preoperative chemotherapy: 26 patients (33.8 %) had chronic hepatitis, 20 (26.3 %) had liver cirrhosis, and 23 (30.3 %) had received preoperative chemotherapy. Only one patient had Child-Pugh class B liver function, and none had Child-Pugh class C liver function. The preoperative indocyanine green retention rate at 15 min was similar in both groups.

**Table 2:** Preoperative characteristics of patients

Characteristic	Group-S (*n* = 20)	Group-SP (*n* = 56)	*P*	Total (*n* = 76)
Age	66 (49–78)	66 (39–86)	NS	66 (39–86)
Sex (M/F)	17/3	45/11	<0.05	62/14
BMI	23.1 ± 2.7	23.6 ± 2.9	NS	23.6 ± 2.7
Previous laparotomy [n (%)]	7 (30.4)	28 (50.0)	NS	35 (46.1)
Preoperative chemotherapy	4 (20.0)	19 (33.9)	NS	23 (30.3)
HBsAg (+) (%)	5 (25.0)	7 (12.5)	NS	12 (15.8)
Anti-HCV AB (+) (%)	9 (45.0)	16 (28.6)	NS	25 (32.9)
Liver disease (normal/CLD/LC)	4/7/9	26/19/11	NS	30/26/20
Child-Pugh class (A/B/C)	19/1/0	56/0/0	NS	75/1/0
ICG-R15	17.9 ± 9.54	15.6 ± 10.7	NS	17.6 ± 10.3

*Group-S* supine patients, *Group-SP* semiprone patients, *NS* not significant, *BMI* body mass index, *HBsAg* hepatitis B surface antigen, *anti-HCV AB* anti-hepatitis C virus antibody, *ICG-R15* indocyanine green retention rate at 15 min

Tumor characteristics are given in Table 3. There were no significant differences in mean tumor size, number of tumors, or location of tumors (surface or deep) between the two groups.

**Table 3:** Tumor characteristics

Characteristic	Group-S (*n* = 20)	Group-SP (*n* = 56)	*P*	Total (*n* = 76)
Size (cm)	3.0 ± 1.3	2.5 ± 1.0	NS	2.9 ± 1.3
Number (1/2/3)	18/2/0	42/13/1	NS	60/15/1
Location (superficial/deep)	19/1	45/11	NS	64/12

*Group-S* supine patients, *Group-SP* semiprone patients, *NS* not significant

The types of liver resection performed are listed in Table 4. The proportions of patients who underwent major resection were not significantly different between the two groups. In patients who underwent minor liver resection, anatomical resections such as S6 and S7 segmentectomy were performed only in the semiprone group (*P* < 0.05). The proportion of nonanatomical resections of S1, S7, and S8 was also significantly higher in the semiprone group than in the supine group (*P* < 0.01).

**Table 4:** Types of laparoscopic liver resection

Type	Group-S (*n* = 20)				Group-SP (*n* = 56)				*P*	Total (*n* = 76)		
Major liver resection	4				14				NS	18		
Right hemihepatectomy (*n*)		2				5					7	
Right posterior sectionectomy (*n*)		2				9					11	
Minor liver resection	17				54				NS	71		
Anatomical liver resection		0				10			<0.05		10	
S6 Segmentectomy (*n*)			0				5					5
S7 Segmentectomy (*n*)			0				3					3
S8 Segmentectomy (*n*)			0				2					2
Nonanatomical liver resection		17				44			NS		61	
S6 Partial resection (*n*)			12				13					25
S1 Partial resection (*n*)												1
S7 Partial resection (*n*)									<0.01			14
S8 Partial resection (*n*)												21
Total number of liver resection	21				68					89		

*Group-S* supine patients, *Group-SP* semiprone patients, *NS* not significant

Intraoperative and postoperative outcomes are given in Table 5. There were no conversions to open surgery in either group. Of the 24 patients with metastatic colorectal cancer, 12 had undergone previous resection of the primary tumor, and 12 underwent simultaneous laparoscopic resection of the primary tumor by low anterior resection (*n* = 5), sigmoid colectomy (*n* = 3), or right colectomy (*n* = 1). One patient in the supine group underwent simultaneous laparoscopic distal gastrectomy for early gastric cancer, which was detected during the preoperative investigation of HCC.

**Table 5:** Surgical outcomes

Outcome	Group-S (*n* = 20)	Group-SP (*n* = 56)	*P*	Total (*n* = 76)
Open conversion	0	0		0
Simultaneous combined resection [n (%)]	3 (15.0)	12 (21.4)	NS	15 (19.7)
Rectum [n (%)]	1 (5.0)	6 (10.7)		7 (9.2)
Sigmoid colon [n (%)]	0	4 (7.1)		4 (5.3)
Right colon [n (%)]	0	1 (1.8)		1 (1.3)
Gastrectomy [n (%)]	1 (5.0)	1 (1.8)		1 (1.3)
Spleen [n (%)]	1 (5.0)	1 (1.8)		1 (1.3)
Operative time (min) (range)	344 (99–685)	296 (66–599)	NS	351 (79–881)
Without simultaneous G-I resection^a^	352 (99–685)	272 (79–578)	NS	
Blood loss (g) (range)	889 (120–3,200)	158 (0–1,070)	<0.05	525 (0–3,200)
Without simultaneous G-I resection^a^	1,101 (120–3,200)	98 (0–350)	<0.05	
Blood transfusion	3 (15.0)	3 (5.4)	NS	6 (7.9)
Postoperative complications [n (%)]	4 (20.0)	2 (3.6)	NS	6 (7.9)
Without simultaneous G-I resection^a^	2 (10.0)	0		
Intra-abdominal abscess [n (%)]	2 (10.0)	2 (3.6)		4 (5.3)
Ascites [n (%)]	1 (5.0)	0		1 (1.3)
Bile leakage [n (%)]	1 (5.0)	0		1 (1.3)
Postoperative hospital stay (days) (range)	35 (7–71)	11 (5–23)	<0.05	21.9 (5–71)
Without simultaneous G-I resection^a^	28 (7–71)	9 (5–14)	<0.05	16.2 (5–71)

*Group-S* supine patients, *Group-SP* semiprone patients, *NS* not significant

Data are presented as median (range) or number (%)

^a^Without simultaneous gastric or colorectal resection

The mean operating time was not significantly different between the semiprone and supine groups. There was less blood loss in the semiprone group (mean 158 g; range 580–1,070 g) than in the supine group (mean 889 g; range 120–3,200 g) (*P* < 0.05), and this difference was greater when patients who underwent simultaneous colorectal or gastric resection were excluded. The mean postoperative hospital stay was shorter in the semiprone group (median 11 days; range 5–23 days) than in the supine group (median 35 days; range 7–71 days) (*P* < 0.05), and this difference was also greater when patients who underwent simultaneous colorectal or gastric resection were excluded.

Six patients (8.6 %) developed postoperative complications, and the complication rate was similar between the two groups. Bile leakage at the cut surface of the remnant liver occurred in one patient with HCC in the supine group. Postoperative symptomatic intra-abdominal fluid collection occurred in one patient with HCC who developed prolonged ascites after minor liver resection, which resolved after administration of diuretics and limitation of water and salt intake. Intra-abdominal abscesses requiring treatment occurred in four patients who underwent simultaneous colorectal resection. In one of these cases, the abscess was adjacent to the partial resection of S8 and was managed by percutaneous drainage and right colectomy. The other patients had undergone low anterior resection, and the abscesses were adjacent to the bowel anastomoses or in the right lower abdomen. No patients required reoperation, and there were no cases of gas embolism, major complication, or perioperative death.

The results of pathological examinations of the surgical specimens are given in Table 6. There were no significant differences between the two groups in tumor-free margin, minimum distance from resection line to tumor tissue, or weight of resected specimens.

**Table 6:** Histopathological data

Parameter	Group-S (*n* = 20)	Group-SP (*n* = 56)	*P*	Total (*n* = 76)
Tumor-free margin [n (%)]	20 (100)	56 (100)	NS	76 (100)
Minimum distance from resection line to tumor tissue (mm) (range)	4 (1–25)	5 (1–30)	NS	4 (1–30)
Weight of resected specimen (g) (range)	142 (7–800)	201 (9–890)	NS	171 (7–890)

*Group-S* supine patients, *Group-SP* semiprone patients, *NS* not significant

## Discussion

Laparoscopic liver resection in the semiprone position has a number of advantages over that in the supine position. First, Rouviere’s sulcus [16, 17], a fissure on the liver to the right of the hilum, is easily visualized immediately after insertion of the laparoscope. This sulcus is open in 78 % of patients and is recognizable in more than 90 % [18]. The liver stays in position because it is attached to the coronary and right triangular ligaments, but other organs such as the transverse colon and small intestine fall to the lower left. The right hepatic hilum is therefore easily exposed by lifting the edge of the liver or gallbladder. The portal pedicles of the anterior and posterior segments and the segmental pedicles of S6 and S7 can easily be ligated for selective occlusion of the blood supply prior to parenchymal transection.

Second, an intercostal port can be used effectively in the semiprone position. When parenchymal transection is performed using only subcostal ports, transection can be performed from only one direction because the forceps cannot reach the portions of the posterosuperior and anterosuperior segments located adjacent to the diaphragm (Fig. 3). Although it is possible to visualize these areas using a flexible scope or a 30° or 45° laparoscope, it is nearly impossible to operate in this area using only subcostal ports. Partial resection of a posterosuperior or anterosuperior tumor is therefore more difficult than hemihepatectomy or sectionectomy. Right hemihepatectomy involves a relatively small dissection plane and can be performed by approaching the liver from the inferior side at the hepatic hilum, from the front of the IVC. An intercostal port gives access to the abdominal cavity from the seventh intercostal space in the anterior axillary line, passing through the thorax and the diaphragm. This port can be used by the right hand of the operator, allowing the surgeon to approach the liver from both the inferior and the superior aspect to perform partial resection or segmentectomy of S7 and S8. Gayet and co-author [19] used an intercostal port to retract the hepatic veins from a lateral approach. However, as the port was on the left side and the patient was in the supine position, this port could not be used for parenchymal division. The technique they described is clearly different to our procedure [20].

Third, the weight of the liver helps to mobilize it. When the coronary and right triangular ligaments are transected, the right lobe naturally falls to the left, leaving a space under the right side of the diaphragm. This is one of the reasons that an intercostal port can be used and enables division of the blood vessels around the IVC without elevation of the liver by an assistant.

Fourth, the irrigation fluids and blood flow to the lower left side of the abdominal cavity and do not interfere with visualization of the operative field [14, 15].

There was less intraoperative bleeding in the semiprone group than in the supine group for several reasons. The semiprone position enables selective vascular occlusion before parenchymal transection, and the posterior segment is positioned higher than the IVC. We also used an innovative device that had a channel for dripping saline at the tip of a surgical instrument. This device can be attached to various endoscopic bipolar scissors or forceps, including bipolar forceps that can be precisely controlled such as the BiClamp, BiCision, and LigaSure. This equipment is used by some liver surgeons for careful hepatectomy via laparotomy, such as harvesting of a living-donor transplant [21, 22]. However, use of this device in laparoscopic surgery has not been well developed. The saline-dripping channel contains compressed saline or is attached to an infusion pump, and the flow rate can be adjusted from a slow drip to a water jet. Saline dripping prevents adhesion of tissues to the cautery blades by reducing the contact between the electrodes and the tissues, and it washes the blood away. This enables fine parenchymal dissection and careful identification and exposure of the portal pedicles. ENSEAL^®^ (Ethicon Endo-Surgery, Cincinnati, OH) is another modern bipolar device that can coagulate tissues by contact with only one of the jaws [23–25]. This jaw can be used to thinly scoop a portion of hepatic parenchyma before picking up the scooped parenchyma with both jaws and then coagulating and cutting it.

The most common indication for laparoscopic liver resection is HCC in patients with underlying liver disease [7]. Resection of metastatic liver cancer is performed with increasing frequency, but many patients with metastatic cancer have liver damage due to previous chemotherapy [26]. If a sufficient surgical margin can be achieved, partial segmental resection is preferable to segmental resection, and segmental resection is preferable to lobectomy.

The use of innovative techniques and devices enables selective hepatic vascular occlusion and parenchymal division in the semiprone position. This allows us to perform partial resection and anatomical resection of the posterosuperior segments of the right liver with minimal bleeding and ischemic injury.

The semiprone position has some inherent disadvantages. When the laparoscope is initially introduced into the abdominal cavity, the positions and relationships of structures are unfamiliar. Rotation of the camera can help to achieve a more familiar orientation. It is important for surgeons learning this technique to familiarize themselves with the visual field and the locations of organs in this position. For tumors of the hepatic dome, resection is performed from the lower aspect to the upper aspect, even when using an intercostal port. Even if the operator is right-handed, parenchymal dissection should be performed mainly using the left hand while supporting the portion of the liver containing the tumor using the right hand.

We experienced one case of the tracheal tube becoming dislodged. Fortunately, this did not have serious consequences because the anesthesiologist responded immediately. Care must be taken to avoid blindness due to prolonged pressure on the eye, as has been reported in patients who underwent esophagectomy in the prone position.

Three surgeons are now performing hepatectomy in the semiprone position at our institution, and the indications for pure laparoscopic hepatectomy have expanded rapidly since the introduction of this technique. The technique is also being used at a number of other institutions in Japan. We believe that use of this technique has resulted in expansion of the indications for laparoscopic resection of tumors in the anterosuperior and posterior segments and has improved outcomes, irrespective of number of surgical experience of surgeon.

In conclusion, introduction of the semiprone position allowed us to perform laparoscopic liver resection in patients with tumors of the anterosuperior and posterior segments, without increasing the proportion of patients undergoing major hepatectomy. This method is safe and minimally invasive and can reduce intraoperative bleeding and shorten the postoperative hospital stay compared with resection in the supine position.

## Electronic supplementary material

Below is the link to the electronic supplementary material.

Supplementary material 1 (MOV 61636 kb)

Supplementary material 2 (MOV 77051 kb)

Supplementary material 3 (MOV 78501 kb)

Supplementary material 4 (MOV 55223 kb)
